# Current Progress in the Role of Ferroptosis in Skeletal Muscle Atrophy

**DOI:** 10.1155/mi/3578349

**Published:** 2026-04-20

**Authors:** Yu Chen, Yaqing Guo, Zibo Zhao, Siqian Yang, Biao Guo, Jian Xu

**Affiliations:** ^1^ Department of Orthopaedics, Xinchang Hospital of Wenzhou Medical University, 312500, Shaoxing, Zhejiang, China; ^2^ Department of Nursing, Huashan Hospital, Fudan University, Shanghai, 200040, China, fudan.edu.cn; ^3^ College of Chemistry and Chemical Engineering, Lanzhou University, 730000, Lanzhou, Gansu, China, lzu.edu.cn; ^4^ School of Basic Medical Sciences, Guizhou Medical University, Guiyang, 550004, China, gmc.edu.cn; ^5^ Department of Orthopaedics, Sports Medicine and Arthroscopy, Fuyang People’s Hospital Affiliated to Anhui Medical University, 236001, Fuyang, Anhui, China

**Keywords:** ferroptosis, iron metabolism, lipid peroxidation, skeletal muscle atrophy, therapeutic targets

## Abstract

Atrophy of skeletal muscles, caused by multiple factors, including ageing, disuse, trauma and systemic diseases, is a major pathological condition that affects physical performance and systemic health by the loss of muscle function; in effect, weakening the whole body and leading to disabling conditions such as sarcopenia, frailty and increased fall risk. First reported in 2012 by Dixon et al., ferroptosis is a newly identified, distinct type of regulated cell death characterised by unique biochemical and morphological features and differs from classic apoptosis and necrosis. One hallmark feature of ferroptosis is its iron‐dependence: excessive free intracellular iron deposits catalyse rapid peroxidation of membrane lipids, and release cytotoxic lipid peroxides, which interfere with cell integrity. In recent years, significant progress has been made in elucidating the roles of ferroptosis‐related pathways in skeletal muscle atrophy. Building on these advances, our review systematizes skeletal muscle atrophy into three significant categories: ageing‐, disuse‐ and systemic disease‐induced atrophy. It carefully explores the involvement of ferroptosis in each of these atrophy models. Moreover, this review identifies and discusses key ferroptosis‐related molecular targets for consideration, aiming to provide insights and possible future directions for developing therapies to treat muscle‐wasting disorders.

## 1. Introduction

Skeletal muscle atrophy is the pathological loss of muscle mass and strength that occurs when protein degradation exceeds protein synthesis [1, 2]. At the histological level, it is characterised by smaller muscle fibres with reduced cross‐sectional area. Clinically, weakness, easy fatigue and impairments in mobility give rise to falls, disability and reduced quality of life in patients with this condition [3]. Muscle wasting is particularly common in older individuals or those with chronic disease. For example, sarcopenia affects on the order of 5%–10% of people over the age of 65. Estimates of global prevalence have suggested anywhere from approximately 8% to 27% of older adults in the world meet criteria for sarcopenia, with substantial global variation [4]. Musculoskeletal diseases, of which sarcopenia is part, afflict some 1.7 billion people worldwide and are the leading cause of years spent in disability [5]. In the ageing population, sarcopenia can significantly increase the risk of falls and fractures [6], reduce their ability to perform activities of daily living [7], and is associated with heart disease, respiratory disease [8] and cognitive impairment; in addition, the disease also leads to motor dysfunction, resulting in a decline in quality of life, loss of independence, long‐term care needs and elevated mortality risk [5]. From an economic perspective, sarcopenia places a significant burden on healthcare systems. The risk of hospitalisation for such patients increases, and the cost of care during hospitalisation also increases accordingly [9, 10]. In the young adult population, existing evidence suggests that approximately 1 in 10 young people suffers from muscle atrophy, and the disease tends to be in young adults, with its onset associated with multiple factors such as metabolic syndrome, insufficient physical activity and malnutrition. Currently, due to the lack of a standardised definition and tools for muscle atrophy in youth, data on its prevalence are still relatively scarce [11]. Thus, skeletal muscle atrophy is a growing public health issue that negatively impacts mobility and metabolic health and carries significant social and economic costs [12].

Whether it originates from the unloading of muscle atrophy has different causes. Disuse and unloading of muscles (e.g., immobilisation, bed rest and microgravity) rapidly cause fibre atrophy. Muscular inactivity causes oxidative stress in muscle fibres and shifts the protein balance toward degradation [13]. Simple extended immobilisation increases reactive oxygen species (ROS), which, in turn, triggers proteolytic pathways and hampers protein synthesis [14]. In ageing (sarcopenia), there is low‐grade inflammation, hormonal changes and mitochondrial decline. These changes in aged muscle promote NF‐κB‐ and FOXO‐mediated activation of proteasomal and autophagy pathways, leading to a gradual loss of muscle in ageing itself [15]. Systemic diseases can result in muscle wasting. In cancer cachexia, chronic inflammation directly inhibits muscle protein synthesis (MPS) and activates the ubiquitin–proteasome and autophagy–lysosome pathways [16]. Similar inflammatory and catabolic signals occur in chronic conditions such as heart failure, chronic kidney disease (CKD) and chronic obstructive pulmonary disease (COPD), all of which are associated with muscle loss. Underlying all of these conditions, oxidative stress is a common final pathway: excess ROS (often arising from mitochondria) will damage cellular constituents and activate atrophic signalling [17–19]. In short, regardless of whether caused by disuse, ageing or disease, muscle atrophy involves increased proteolysis, a decrease in protein synthesis, chronic inflammation, mitochondrial dysfunction and redox imbalance.

Ferroptosis is a recently characterised type of regulated cell death that is biochemically and morphologically distinct from apoptosis or necrosis [20]. It is defined by iron‐dependent lipid peroxidation: excess free iron drives the metabolism of lethal lipid peroxides of membrane lipids [21]. ‘Ferroptosis’ is the term coined by Dixon and Olzmann [22] in 2012, who reported that small molecules such as erastin could kill cells selectively via an iron‐ and lipid‐dependent ROS‐mediated process. The primary regulatory pathways dovetail onto two axes. First, cellular uptake of cystine by the antiporter System X_c_^–^ (SLC7A11/xCT) is required to synthesise glutathione (GSH). Inhibition of SLC7A11 deprives cells of cysteine, consumes GSH, and thus inactivates the lipid‐peroxide‐detoxifying enzyme GSH peroxidase 4 (GPX4). GPX4 normally converts lipid hydroperoxides to a non‐toxic alcohol using GSH; inhibition of GPX4 leads to unregulated lipid peroxidation and the initiation of ferroptosis [23]. Second, iron metabolism genes alter susceptibility. Ferritin heavy chain (FTH1) stores iron safely; loss of ferritin leads to the release of labile iron and amplifies lipid oxidation. Iron can also enter across transferrin receptors (Tfrs) or divalent transporters, fuelling Fenton chemistry in membranes [24].

Many studies have already implicated ferroptosis in the development of various human diseases. In cancer, ferroptosis can suppress tumours, and many anticancer therapies induce ferroptosis in tumours [25–28]. In neurodegeneration, lipid‐ROS‐induced cell death is strongly implicated: models of Parkinson’s, Alzheimer’s and Huntington’s disease exhibit neuronal protection with ferroptosis inhibitors (ferrostatin‐1 and liproxstatin‐1) [29, 30]. For example, ferroptosis cardiomyocyte loss in cardiovascular disease results in ischaemia/reperfusion injury, heart failure and cardiomyopathy [31, 32]. Both myocardial infarction and doxorubicin cardiotoxicity are characterised by GPX4 downregulation and iron accumulation, and experimental blockers of ferroptosis decrease infarct size. Overall, dysregulated ferroptosis (too much or too little) is associated with cancer, neurodegeneration, cardiovascular disease and many forms of tissue injury. These results also identify ferroptosis as an important cell‐death pathway in disease, and many studies demonstrate that ferroptosis inhibitors can prevent disease in animal models.

Emerging evidence also suggests that ferroptosis is involved in skeletal muscle atrophy. Several recent reports point to an iron overload and lipid peroxidation in atrophying muscle. For example, aged rodents and humans with sarcopenia have higher muscle iron content and ferritin accumulation [33]. Atrophic muscles exhibit excess iron loading, disrupting signalling processes (decreased AKT/FOXO3a phosphorylation) and inducing oxidative stress, which drives protein breakdown [34]. What we mean by iron‐mediated lipid peroxidation is that it leads to ferroptosis, which disturbs muscle homeostasis and promotes sarcopenia much more rapidly [35].

Ferroptosis also impairs skeletal muscle repair. Muscle stem cells are sensitive to iron dysregulation. Genetic deletion of the Tfr1 in satellite cells induces mitochondrial dysfunction and iron overload and causes spontaneous ferroptosis and loss of regenerative function. Studies of muscle injury models showed that iron‐loaded satellite cells showed delayed proliferation and differentiation and reduced muscle regeneration. Treatment with a ferroptosis inhibitor (ferrostatin‐1) in these models partially restored satellite cell function and improved muscle repair [36]. Together, these findings suggest that iron overload, mitochondrial ROS and lipid peroxidation occur in atrophic muscle and its progenitor cells, and that ferroptosis is linked to the progression of muscle wasting.

Having considered these insights, ferroptosis is emerging as a new mechanism of muscle atrophy, offering new targets for therapeutic intervention. Metabolic pathways associated with ferroptosis, such as iron metabolism, GSH metabolism and lipid peroxidation, have now been shown to be involved in skeletal muscle atrophy, cardiac muscle injury and motor neurone degeneration. Targeting ferroptosis thus paves promising strategies for preventing or treating muscle‐wasting disorders. Our aim with this review was to assimilate recent discoveries about ferroptosis in skeletal muscle atrophy: to summarise evidence of the involvement of iron and lipid‐peroxidative death in muscle loss; to review the underlying molecular mechanisms; and to discuss the fact that ferroptosis can guide new therapies for muscle atrophy.

The search strategy adopted for the review is as follows: the first two authors conducted independent searches in December 2025 using three major databases: PubMed, Embase and the Cochrane Library. Search terms were formulated based on previous relevant reviews and professional consultations with a specialist librarian. The search strategy incorporated the following keywords: ‘Ferroptosis’, ‘Muscle Atrophy’, ‘Sarcopenia’ and ‘Muscular Atrophies’. Additionally, the reference lists of all retrieved relevant studies were manually reviewed to identify any potentially eligible studies that might have been missed in the initial database search.

## 2. Overview of Ferroptosis

### 2.1. Definition and Characteristics of Ferroptosis

Ferroptosis is a new type of programmed cell death, with dependence on iron and lethal lipid peroxidation [37]. Since its original identification in 2012, ferroptosis has been defined as radically different from apoptosis, necrosis and other programmed cell death mechanisms in morphology, biochemistry and genetic regulation [22]. Like apoptosis, however, ferroptosis does not lead to caspase activation, chromatin condensation or apoptotic body formation; nor does it have the characteristic features of rapid plasma membrane rupture or inflammation of necrosis. Instead, ferroptosis is driven by an imbalance between oxidative damage and antioxidant defence, leading to the uncontrolled accumulation of lipid hydroperoxides within cell membranes [38].

At the morphological level, ferroptosis cells generally exhibit intact nuclear morphology and profound mitochondrial abnormalities [39]. These include a smaller mitochondrial volume, a higher density of the mitochondrial membrane, loss or condensation of cristae and the rupture of the outer mitochondrial membrane. Such ultrastructural changes illustrate the central contributions of mitochondria and membrane lipid composition in ferroptosis and related cell death. Biochemically, ferroptosis is defined by excessive iron‐dependent ROS formation and lipid peroxidation of polyunsaturated fatty acid (PUFA)‐containing phospholipids. Due to the redox‐active Fe^2+^, a Fenton reaction occurs, increasing oxidative stress and aiding the propagation of lipid peroxyl radicals, which eventually damage the membrane [40].

One characteristic of ferroptosis is its exclusive dependence on iron levels and lipid peroxidation, but not on protein or DNA damage. Cells undergoing ferroptosis display highly elevated levels of the lipid peroxidation product groups malondialdehyde (MDA), 4‐hydroxynonenal (4‐HNE) and depletion of intracellular GSH, as well as loss of activity of the lipid repair enzyme GPX4 [41]. Importantly, ferroptosis can be specifically prevented by iron chelators or lipid radical‐trapping antioxidants, such as ferrostatin‐1 and liproxstatin‐1, emphasising its distinct mechanism from other forms of cell death [42].

Functionally, ferroptosis is a context‐dependent phenomenon that has both pathological and (potentially beneficial) roles. Acute ferroptosis may promote the removal of severely damaged or poorly metabolised cells, but sustained, aberrant ferroptosis signalling pathways result in tissue degeneration and the chronic progression of disease [43]. As skeletal muscle is rich in mitochondria, iron and oxidisable lipids and is a constant target of metabolic and oxidative stress, ferroptosis represents a critical contributing pathway in muscle disorders, such as muscle atrophy.

### 2.2. Molecular Regulatory Networks Governing Ferroptosis

Regulated by a complex network that interconnects iron metabolism, lipid remodelling, redox homeostasis and stress signalling pathways, ferroptosis [44]. At the heart of this network lies the balance between lipid peroxide formation and the cellular machinery that degrades these peroxidized lipids.

The canonical protective pathway towards ferroptosis is the System X_c_^–^/GSH/GPX4 axis [45, 46]. System X_c_^–^ is a cystine/glutamate transporter formed by a light chain subunit (SLC7A11; xCT) and the heavy chain SLC3A2, and imports cystine into cells. Once imported, cystine is reduced into cysteine, the limiting substrate for GSH synthesis. GSH, in turn, is a limiting cofactor for GPX4, the enzyme that catalyses the reduction of phospholipid hydroperoxides into non‐toxic lipid alcohols. Once System *X*_c_^–^ has been inhibited, intracellular GSH has been depleted, or GPX4 has been directly inactivated, the lipid peroxidation becomes unchecked, and cells undergo ferroptosis [47]. It is for this reason that SLC7A11 and GPX4 have been considered key sensors of ferroptosis.

However, lipid metabolism is another determinant of the extent of ferroptosis [29]. Not all membrane lipids are equal opportunities for peroxidation; rather, ferroptosis preferentially affects phospholipids with PUFAs. Another example is the long‐chain acyl‐CoA synthetase family member 4 (ACSL4). These enzymes are key to the incorporation of PUFAs, such as arachidonic acid and adrenic acid, into membrane phospholipids [48]. These PUFA‐enriched phospholipids become subsequent substrates for both enzymatic and nonenzymatic oxidation, including enzymatic catalysis by lipoxygenases (ALOXs). Expression or activity of ACSL4 and LPCAT3 increases membrane vulnerability to lipid peroxidation and sensitises cells to ferroptosis, whereas inhibition has a protective effect.

Iron homeostasis is the third indispensable aspect of ferroptosis control [49]. Cellular iron uptake is mediated by Tfr1, a receptor that mediates transferrin‐bound iron internalisation. Once inside the cell, iron can be safely sequestered in ferritin complexes, which comprise the ferritin heavy and light chains. A selective form of autophagic destruction of ferritin, termed ferritinophagy, mediated by nuclear receptor coactivator 4, releases iron to the labile iron pool. Excess labile iron leads to ROS formation and rapidly promotes lipid peroxidation, amplifying the ferroptosis programme. Poor regulation at any step in the iron‐handling process can prime a cell for ferroptosis.

In addition to the earlier‐described basic pathways, several context‐specific regulators of ferroptosis sensitivity exist. The transcription factor nuclear factor erythroid 2‐related factor 2 (NRF2) is a master regulator of antioxidant and cytoprotective genes [50, 51]. Activation of NRF2 induces expression of SLC7A11, GPX4, ferritin and synthesis‐related enzymes for the reactive thiol GSH, collectively boosting resistance to ferroptosis. Conversely, the tumour suppressor p53 can stimulate ferroptosis under favourable conditions by repressing SLC7A11 transcriptionally, downregulating cystine uptake and weakening the antioxidant machinery. Finally, note that p53‐activated ferroptosis is highly context‐ and cell type‐dependent.

Recent studies have also revealed GPX4‐independent anti‐ferroptosis protection pathways [52]. Ferroptosis suppressor protein 1 reduces coenzyme Q10 to its antioxidant form, ubiquinol, which scavenges lipid radicals at cell membranes. Similarly, dihydroorotate dehydrogenase operates in mitochondria to maintain CoQH_2_ levels and protect against mitochondrial lipid peroxidation. Such parallel pathways reflect redundancy and robustness in the cellular anti‐ferroptosis network.

Collectively, ferroptosis is regulated by an integrated molecular machine comprising antioxidant capacity, lipid content, iron availability and stress‐signalling. Disruption of this balance, particularly in high‐metabolic‐demand, highly oxidative‐tolerant tissues, such as skeletal muscle, would presumably make cells more prone to ferroptosis. Understanding these regulatory networks lays the foundation for exploring ferroptosis in muscle atrophy and for identifying targeted therapies.

## 3. Ferroptosis in Skeletal Muscle Atrophy

### 3.1. Ageing‐Related Skeletal Muscle Atrophy

Ageing‐related skeletal muscle atrophy, also known as sarcopenia, is a degenerative disease characterised by a gradual decline in muscle mass, strength and function. It is closely associated with an increased risk of falls, fractures and mortality in older adults [53, 54]. Studies have also demonstrated that sarcopenia affects about 10%–16% of the elderly population worldwide. Differences in the prevalence of sarcopenia result primarily from differences in diagnosis standards and assessment techniques in different places [55]. The pathogenesis of sarcopenia is complex and multifactorial, including decreased physical activity, hormonal alterations, malnutrition, inflammatory states and oxidative stress [33]. More attention is being paid to the role of ferroptosis, a form of regulated cell death (dependent on iron and lipid peroxidation), in the pathogenesis of sarcopenia in recent years [56].

Recent investigations indicate that ferroptosis occurs in association with disturbances in iron homeostasis in skeletal muscle tissue. Around 60% of the non‐haem iron in the human body is stored in muscle tissue [57]. With ageing and worsening of iron dysmetabolism and abnormal iron deposition as non‐haem iron, ROS generated by Fenton reactions accumulate and lipid peroxidation increases, leading to ferroptosis of muscle fibres [58]. Multiple regulatory molecular pathways regulate this process. Using ageing animal models, such as the 40‐week‐old senescence‐accelerated mouse prone 8 (SAMP8), studies have shown dramatically elevated levels of iron and ferroptosis‐related markers in the muscle tissue of aged animals [59]. These include upregulation of p53, which transcriptionally silences crucial genes, such as solute carrier family 7 member 11 (SLC7A11), and, more generally, downregulation of proteins, such as FTH1, GPX4 and the cystine/glutamate transporter. Collectively, these molecular changes lead to the loss of antioxidant defences and the development of toxic products of lipid peroxidation, such as lipid hydroperoxides (LOOH) [59, 60]. Other regulatory pathways participate in this process; for example, the activating transcription factor (ATF3) suppresses ferroptosis in muscle cells and directly activates the PI3K/Akt signalling pathway [61]. Simultaneously, the FOXO1/thioredoxin‐binding protein 2 (TXNIP) axis, which promotes ferroptosis via disruption of GSH metabolism, was also identified as a promoter of ferroptosis [62]. Such complex interactions further complicate the regulation of ferroptosis in the context of muscle atrophy.

Iron accumulation and ferroptosis have adverse effects not only on mature muscle fibres but also severely diminish the regenerative function of skeletal muscle [63]. Muscle satellite cells are nestled underneath the basal lamina of muscle fibres and are essential for muscle repair and regeneration. Excessive iron severely impairs the self‐renewal and regenerative function of these cells by downregulating the satellite cell marker and inhibiting satellite cell proliferation and differentiation [64, 65]. For example, dysfunction of Tfr1 worsens iron accumulation and mitochondrial dysfunction in ageing satellite cells and restricts skeletal muscle regeneration through ferroptosis, aggravated by iron accumulation in aged muscle [66]. These findings together demonstrate that iron accumulation‐induced ferroptosis in muscle cells and satellite cells is critical, acting as a pivotal step that disrupts muscle homeostasis and promotes the progression of sarcopenia.

It should be noted that the complex interplay between ferroptosis and mitochondrial dysfunction contributes to sarcopenia pathogenesis. However, in addition to their essential role in ferroptosis, mitochondria can be affected in function and structure by disturbances in iron metabolism [67]. Ageing skeletal muscle also frequently presents an imbalance in mitochondrial dynamics with reduced abundance of the fusion‐promoting protein Mitofusin 2 (Mfn2) and inactivation of the fission regulator dynamin‐related protein 1 (Drp1). However, these disturbances impair mitochondrial autophagy, leading to the accumulation of defective mitochondria [68–72]. Also, we observed downregulation of essential mitochondrial autophagy proteins, including E3 ubiquitin ligases, further reducing the efficiency of cellular quality control [73]. Direct evidence in humans supports these findings. For example, old human skeletal muscle was found to have elevated iron levels, along with elevated mitochondrial DNA (mtDNA) copy number and more‐early damage, both of which are indicative of iron metabolism abnormalities that may interfere with mitochondrial genomic stability and function due to oxidative damage [74]. His mitochondrial dysfunction and ferroptosis form a vicious cycle that is collectively very deleterious to muscle atrophy. Some authors speculated that ferroptosis may sometimes be protective, turning on to purify severely damaged cells [75]. Still, the net effect of ferroptosis in the chronic progression of sarcopenia is predominantly harmful [76, 77].

Given these mechanisms, researchers are exploring various intervention strategies to overcome these problems. Exercise has shown promise too, especially regular aerobic exercise training, which upregulates expression of superoxide dismutase 2 (SOD2), modulates inflammatory factors, such as leptin and TNF‐α, and alleviates oxidative stress, inflammation and ferroptosis [60]. High‐intensity interval training, together with nutritional supplements such as glycine, has also shown potential to increase muscle mass through modulating mitochondrial function in animal models [78]. There are also pharma‐like products, such as Irisin and ellagic acid (ECH), from traditional Chinese medicine, that show remarkable potential for anti‐ferroptosis and antioxidant properties [56, 79]. Furthermore, environmental factors (e.g., chronic heat exposure) have been recognised as influencing muscle health; they may also disturb the balance of the gut microbiota and their metabolites, thus introducing new risk factors that exacerbate sarcopenia [80]. Collectively, these studies yield novel insights and promising targets for the prevention and therapy of sarcopenia, connecting basic research to the most practical aspects of clinical practice.

### 3.2. Disuse‐Related Skeletal Muscle Atrophy

Disuse‐related skeletal muscle atrophy is the specific process by which skeletal muscle progressively loses mass and function over time through immobilisation, decreased mechanical loading, prolonged bed rest, limb immobilisation or simulated microgravity conditions [81]. Skeletal muscle is a highly plastic tissue, and the amount of muscle mass and muscle phenotype are critically determined by mechanical loading stimuli [82, 83]. It is fuelled by the sedentary lifestyle we lead nowadays, with over 80% of adults failing to meet the recommended level of physical activity. In older adults, more than 67% engage in sedentary behaviour for more than 8.5 ha a day, increasing their risk of muscle atrophy by a large margin [84, 85].

On the molecular level, the essential link of the disuse muscle atrophy is the disruption of the dynamic balance between MPS and muscle protein breakdown (MPB). And in cases of disuse atrophy in humans, the rate of MPB usually does not change significantly; in such cases, the net loss of muscle protein is due primarily to a drastic reduction in MPS [86, 87]. But this metabolic disturbance is accompanied by a cascade of ongoing cellular stresses that can become entangled, such as oxidative stress, mitochondrial dysfunction, chronic inflammation, dysregulated autophagy and apoptosis [88]. Recently, ferroptosis (a regulated cell‐death response to oxidative stress that depends on iron and lipid peroxidation) has been increasingly implicated as a contributing factor in disuse‐induced muscle atrophy [89].

Several preclinical studies provide substantial evidence for a role of ferroptosis in disuse‐mediated skeletal muscle atrophy. While already an established model in animals for conditions of disuse or immobilisation (hindlimb suspension), researchers used this model to identify features characteristic of ferroptosis in atrophied skeletal muscle. That is the characteristic of each of these phenotypes, such as abnormal iron accumulation in tissues, lower activity and expression of the major antioxidant protein GPX4, low expression of the cystine/glutamate exchanger (xCT/SLC7A11) and significantly increased concentrations of lipid peroxidation products [89, 90]. Comprehensive analysis of metabolomic and proteomic pathways reveals systematic disruptions in iron metabolism and antioxidant defence pathways in disuse‐atrophied muscles. Notably, upregulation of ferritin, metal reductase six‐transmembrane epithelial antigen of the prostate 3 (STEAP3) and nicotinamide adenine dinucleotide phosphate (NADPH) oxidase 2 (NOX2) was observed, along with a decline in GSH metabolism, collectively leading to a microenvironment that favours ferroptosis [56]. Lipid peroxidation is indeed critical in this process, as the accumulation of LOOH is a crucial participant in muscle atrophy [91]. Targeted research in this pathway shows, for instance, that inhibiting lysophosphatidylcholine acyltransferase 3 (LPCAT3), a key enzyme in the Lands cycle, can prevent intermembrane spread of LOOH and, therefore, substantially slow the progression of disuse muscle atrophy, and uncover significant new targets for treatment [92].

Disuse can lead to muscle atrophy, including the direct atrophy of muscle fibres. Disuse‐induced muscle loss often accompanies a pathological condition called intramuscular fat infiltration (IMFI). These studies have shown that this event is primarily linked to the inappropriate activation and differentiation of fibro/adipogenic progenitor cells (FAPs) in the muscle stroma into adipocytes [93]. Despite the reported association between IMFI and impaired muscle function, how exactly it develops and is regulated in such purely disuse settings remains to be elucidated [56].

With an understanding of the earlier mechanisms, especially of the ferroptosis pathway, new interventions are currently under development. For instance, the naturally occurring amino acid taurine protects skeletal muscle against disuse atrophy in model systems by effectively inhibiting ferroptosis [89]. These results support the idea that drugs or nutritional supplements targeting the key nodes of ferroptosis (e.g., iron metabolism, GPX4 regulation and lipid peroxidation) could be a possible avenue for preventing or treating muscular disease.

### 3.3. Skeletal Muscle Atrophy With Systemic Diseases

Atrophy of muscle is a common and widespread condition encountered in many different systemic diseases, such as cancer cachexia, diabetes, CKD, sepsis and obesity, with significant consequences for patients’ quality of life and prognosis [18]. Traditionally, the pathogenesis of muscle atrophy has been explained as driven by an imbalance between protein synthesis and degradation; however, this model cannot account for the rapid muscle loss observed in some pathological conditions. There have recently been essential insights into ferroptosis, a new form of programmed cell death that is iron‐dependent and induced by lipid peroxidation, as a molecular pathogenic link between many disease states and muscle wasting. According to this emerging view, new ideas and approaches are emerging to explain and intervene in muscle atrophy [54, 94]. Present evidence points to the importance of ferroptosis in muscle atrophy across various systemic disease contexts. Currently, in the case of oncology, it is known that tumours trigger muscle ferroptosis by ‘remote communication’ of tumours. For example, long non‐coding RNAs secreted by lung cancer exosomes or miR‐203a‐3p secreted by pancreatic cancer lead to GPX4 downregulation in muscle tissues, accelerating the cachexia process [95, 96].

Another effect of chemotherapeutics, such as cisplatin on muscles is intimately tied to the direct induction of ferroptosis, in which they may, in part, be responsible for programmed cell death receptor 1. Using ferroptosis inhibitors, such as dihydromyricetin, also demonstrates protection in this setting [97, 98]. Factors driving ferroptosis in metabolic disorders, such as those induced by oxidative stress and mitochondrial dysfunction, including high glucose, a high‐fat diet or both, contribute to conditions propitious for ferroptosis. For example, in models of sarcopenic obesity induced by a high‐fat diet, activation of interferon‐induced gene‐signature pathways is an upstream signal for ferroptosis. Conversely, in physical exercise, there was a protective effect on fitness and/or bone cells by blocking these interferon‐induced gene‐signature pathways [94, 99]. Further to differences in expression of the BOLA3 protein within diabetic muscle, as well as work using deuterated PUFAs (D‐PUFAs) to increase lipid membrane resistance to peroxidation, the evidence is convincing that lipid peroxidation plays a role in this process [99, 100]. Other endocrine disorders (for example, oestrogen deficiency) could also exacerbate muscle atrophy, at least in part, through mechanisms underlying ferroptosis. However, natural products such as luteolin have also shown promise in inhibiting ferroptosis [101].

In the field of CKD, ferroptosis has now been recognised as a critical and actionable therapeutic target. Research on an adenine‐induced CKD rat model showed iron overload and downregulation of the antioxidant GPX4 in the muscle, representative features of ferroptosis [102]. Natural compounds such as diosgenin and caffeic acid, as well as traditional Chinese medicine compounds in ‘Shenshuai Yingyang Capsule’, can reverse muscle atrophy by inhibiting the ferroptosis pathway [103, 104]. These same pathogenic features are also seen in other chronic inflammatory diseases, such as osteoarthritis, where senescent macrophages can induce ferroptosis in resident muscle cells via paracrine signalling. Furthermore, changes in markers of ferroptosis in muscle biopsies from patients with peripheral artery disease have underscored the importance of local inflammation and ischaemia in the ferroptosis process [36, 105]. In acute critical conditions, such as sepsis, severe systemic inflammation and oxidative stress, muscle ferroptosis is identified as the leading cause. Recent studies have shown that the antioxidant edaravone improves diaphragm function in sepsis by activating the sirtuin 1 (SIRT1)/NRF2) pathway. At the same time, inhibition of signal transducer and activator of transcription 6 (STAT6) reduces muscle atrophy, which is associated with ferroptosis regulation [106, 107]. On the other hand, diseases such as severe acute pancreatitis can systematically induce skeletal muscle ferroptosis by organ crosstalk across the axis between ’pancreas’ and ’muscle’ in the human body. There is also fundamental research implicating FTH1 and the mammalian target of rapamycin complex 1 (mTORC1) signalling pathway in the regulation of iron metabolism and ferroptosis [108–110].

Research in this area is multidisciplinary, encompassing clinical analyses of muscle biopsy samples from patients, bioinformatics methods for identifying key genes and pathways, and the development of various animal and in vitro cell models for mechanistic validation. Hence, all of these constitute a full range of approaches that link clinical association with the identification of causal mechanisms [95, 105, 107]. There is consensus in the academic community that ferroptosis is an important mechanism contributing to muscle atrophy in many systemic diseases and that oxidative stress is a common upstream factor. However, there are still considerable controversies and limitations. Central points of debate include whether there is a single, centralised hub that governs ferroptosis induction, or whether there are parallel, decentralised pathways, and how ferroptosis interacts with known atrophy pathways (such as autophagy and apoptosis). Besides, current research faces several limitations: inadequate systematic consideration of the crosstalk among different organs; over‐reliance on animal models and cell lines has made it difficult to identify more non‐invasive clinical biomarkers; and the extent to which ferroptosis manifests differently in different muscle fibres, its function in muscle regeneration [54, 111, 112].

## 4. Therapeutic Potentials of Ferroptosis‐Related Treatments

### 4.1. Pharmacological Agents

Notably, several small‐molecule inhibitors that rescue preclinical models of muscle atrophy interfere with ferroptosis. Prototypical inhibitors, such as ferrostatin‐1 and liproxstatin‐1, are lipophilic radical‐trapping antioxidants that inhibit the propagation of lipid peroxyl radicals [91, 113]. In cultured myotubes, ferrostatin‐1 fully inhibited lipid hydroperoxide accumulation and rescued atrophy induced by GPX4 knockdown or system X_c_^–^ inhibition [45]. In vivo, in denervation (and disuse) models, treatment with liproxstatin‐1 largely ameliorated markers of ferroptosis, inhibiting lipid peroxide accumulation and maintaining muscle mass and force [114]. Similarly, administering ferrostatin‐1 also improved survival and attenuated rhabdomyolysis in mice subjected to exertional heat stroke, an ACSL4‐induced model of ferroptosis [115].

Other pharmacologics act upstream. Iron chelators (such as deferoxamine, deferiprone or dexrazoxane) regulate the labile iron pool and attenuate ferroptosis‐induced cell death in muscle cells [116]. These agents limit Fe^2+^‐mediated ROS generation and, by extension, indirectly limit lipid peroxidation. Antioxidant supplements also have ferroptosis‐modulating activity: for example, the GSH precursor N‐acetylcysteine (NAC), and ubiquinol (CoQ_10) derivatives that mimic GPX4. Cell assays showed that N‐acetylcarnosine worked as ferrostatin did by scavenging lipid radicals and preventing cell death [117]. In general, these chemical strategies are viewed as ways to reinforce the natural endogenous anti‐ferroptotic pathway or to scavenge ferroptosis‐related toxic products.

Despite exciting preclinical results, translating them into clinical use poses challenges. To date, neither ferrostatin‐1 nor liproxstatin‐1 is FDA‐approved for use in humans [91]. These drugs have short half‐lives and poor safety profiles. Several DFO and related chelators are approved for conditions such as iron overload, but prolonged systemic chelation has numerous side effects and may not be muscle‐specific. To date, no small‐molecule ‘GPX4 activator’ has reached the clinic. Most importantly, achieving effective concentrations in skeletal muscle will be difficult, as will avoiding off‐target effects. Therefore, although pharmacological ferroptosis inhibitors have promise for the treatment of conditions such as cachexia or disuse atrophy, work needs to be done to understand dose, delivery and toxicity before clinical trials.

### 4.2. Natural Compounds

Some endogenous or dietary components have been identified that modulate ferroptosis in beneficial ways for muscle. Taurine is a sulphur‐containing amino acid that prevents disuse muscle atrophy. Overall, in a mouse hindlimb suspension model, taurine supplementation restored muscle mass and grip strength and reduced ferroptosis markers, lowering iron deposition and lipid peroxidation and rescuing levels of GSH, GPX4, NRF2 and xCT [89]. Metabolomic analysis showed that taurine acted through both the xCT–GSH–GPX4 axis and an AMPK–ACC–ACSL4 lipid pathway. The findings suggest that taurine exerts antioxidant effects and prevents ACSL4‐mediated peroxidation, thereby limiting muscle damage.

Myokine irisin has also been studied. In aged mice with sarcopenia, daily irisin injections improved muscle function and size in a dose‐dependent manner. Mouse muscle analyses showed that irisin reduced iron load and restored GPX4 and SLC7A11 expression, accompanied by increased SIRT1 activity and decreased p53 activation [79]. This result suggests that irisin induces resistance to ferroptosis, thereby improving age‐induced atrophy.

Metabolic supplements, when coupled with exercise, can also help. A recent study found that HIIT combined with dietary glycine was beneficial in aged mice, strengthening muscle mass and function by improving ferroptosis defence. Supplementing mice with glycine was associated with higher GSH levels and lower lipid‐ROS levels in muscle, thereby enhancing anti‐ferroptosis capacity. Conceptually, providing amino acid precursors boosts GSH synthesis, and exercise upregulates antioxidant mechanisms.

Traditional herbal remedies were also tested. For example, ‘Shenshuai Yingyang Capsule’ is a multi‐herb Chinese medicine, which has been shown to prevent skeletal muscle atrophy in rats with CKD by suppressing ferroptosis [104]. ‘Shenshuai Yingyang Capsule’ decreased tissue iron and MDA levels and elevated GSH, NADPH and GPX4 levels. Analysis of network implicated that the treatment induced HIF‐1α signalling, which upregulated SLC7A11 to enhance cystine uptake. Other natural antioxidants, such as carnosine, vitamin E and curcumin, could act in much the same way by trapping lipid aldehydes or strengthening endogenous defences too, but trials in muscle are sparse.

### 4.3. Physical Interventions

Effects of lifestyle and physical modalities on muscle ferroptosis‐related pathways. Exercise training itself exerts protective antioxidant effects: resistance and aerobic exercise induce NRF2 and downstream enzymes, which inhibit ferroptosis. For instance, HIIT (see note above) was associated with increased GSH/GPX4 in muscle. By contrast, inactivity (disuse or denervation) aggravates the build‐up of iron and lipid peroxide in the muscle, suggesting that exercise has a protective effect.

Thermal interventions also have complex effects. Controlled heat stress induces heat shock proteins that antagonise ferroptosis. Specifically, high levels of heat‐shock protein beta‐1 (HSPB1, also called HSP27) inhibit ferroptosis by suppressing TFRC‐mediated iron uptake [118]. In principle, mild heat therapy (e.g., sauna or locally heated environment) may upregulate HSP and antioxidant systems, thereby protecting the muscle from ferroptosis. Conversely, excessive heat is a threat: exertional heat stroke triggers ACSL4‐induced lipid peroxidation in skeletal muscle, leading to acute rhabdomyolysis [115]. In a mouse model of heat stroke, ACSL4 inhibition or ferrostatin‐1 dramatically increased survival and prevented muscle necrosis. These findings show that even moderate heat/HSP induction confers resilience, whereas extreme heat triggers ferroptosis in muscle. Thus, any ‘thermo‐therapy’ would have to be dosed carefully.

Other physical strategies (such as mechanical loading or vibration) may also indirectly modulate ferroptosis by altering muscle metabolism, although such studies are scarce. However, translation into humans is a work in progress. Many interventions that show promise (such as ferrostatins and liproxstatin‐1) are not approved for clinical use and are likely to have poor bioavailability. Natural supplements, such as taurine and glycine are safe, but effective doses that deliver into muscle tissue must be defined. Herbal formulas contain complex mixtures and are difficult to standardise and to regulate for approval. However, few clinical trials report ferroptosis‐specific biomarkers.

In summary, targeting ferroptosis offers a novel avenue for treating muscle atrophy. Most evidence is preclinical, however. Further work is needed to optimise these therapies, establish safety/efficacy in humans and develop reliable biomarkers before ferroptosis‐modulating treatments will be widely used in the clinic.

## 5. Conclusion

Such ferroptotic skeletal muscle atrophy plays an important role in diverse causes of skeletal muscle atrophy, such as ageing, disuse and systemic diseases. More central to this process are iron overload, GPX4 dysfunction and lipid peroxidation, all of which can increase oxidative stress and cause protein damage. Preclinical interventions to counter ferroptosis (radical‐trapping antioxidants, iron chelators, exercise, for example) have the potential to preserve muscle mass and function. However, several challenges remain to be addressed; for instance, improving drug delivery, identifying appropriate biomarkers for clinical monitoring and addressing crosstalk with other cell death pathways, such as apoptosis and autophagy. Future research directions should primarily include: (1) deepening the study of the cross‐regulatory mechanisms between ferroptosis and other programmed cell death pathways; (2) focusing on clinical translational research to overcome existing limitations and advance the clinical application of ferroptosis regulatory strategies; (3) exploring the upstream molecular mechanisms and microenvironmental regulatory networks governing ferroptosis; (4) investigating specific regulatory mechanisms of ferroptosis in muscle atrophy within special populations and scenarios; enhancing interdisciplinary research to drive innovative breakthroughs in ferroptosis and muscle atrophy studies.

In summary, as a pivotal component in the pathogenesis of muscle atrophy, the in‐depth exploration of ferroptosis regulation and its clinical translation holds significant implications for the precise diagnosis, effective treatment and improved prognosis of muscle atrophy. Future efforts should focus on the aforementioned research directions, address existing limitations, deepen the investigation of the relationship between ferroptosis and muscular atrophy and advance the translation of basic research findings into clinical applications. Ultimately, this will provide novel approaches and strategies for the prevention and treatment of muscular atrophy, enhance patients’ quality of life and alleviate the public health burden.

## Author Contributions

Conceptualisation: Biao Guo and Jian Xu. Methodology, writing – original draft: Yu Chen and Yaqing Guo. Software, writing – original draft preparation: Zibo Zhao and Siqian Yang. Reviewer: Biao Guo and Jian Xu.

## Acknowledgments

The authors have nothing to report.

## Funding

The authors declare that no funds, grants or other support were received during the preparation of this manuscript.

## Disclosure

All the authors have read and approved the final manuscript.

## Ethics Statement

The authors have nothing to report.

## Consent

The authors have nothing to report.

## Conflicts of Interest

The authors declare no conflicts of interest.

## Data Availability

The authors have nothing to report.
